# Effects of genetic variations in the Adiponectin pathway genes on the risk of colorectal cancer in the Chinese population

**DOI:** 10.1186/1471-2350-12-94

**Published:** 2011-07-12

**Authors:** Bangshun He, Yuqin Pan, Ying Zhang, Qian Bao, Liping Chen, Zhenlin Nie, Ling Gu, Yeqiong Xu, Shukui Wang

**Affiliations:** 1 The Central Laboratory of Nanjing First Hospital Affiliated to Nanjing Medical University, Nanjing 210006, Jiangsu Province, China; 2 Clinical Laboratory, Traditional Chinese Medicine Hospital of Kunshan, Kunshan 215300, Jiangsu Province, China; 3 Department of Life Sciences, Nanjing Normal University, Nanjing 210046, Jiangsu Province, China

**Keywords:** Colorectal cancer, Polymorphism, *ADIPOQ*, *ADIPOR*, Genetic susceptibility

## Abstract

**Background:**

Decreased expression of adiponectin (*ADIPOQ*) is associated with an increased risk for developing colorectal cancer (CRC) in humans. This study was designed to determine whether polymorphisms present in the *ADIPOQ *and its type 1 receptor (*ADIPOR1*) could affect the risk of CRC.

**Methods:**

We measured five polymorphisms in the *ADIPOQ *and two polymorphisms in *ADIPOR1*, and analyzed their associations with CRC risk in 420 CRC patients and 555 age- and gender-matched healthy individuals.

**Results:**

Multivariate logistic regression analyses revealed that the CRC risks (adjusted odds ratio and 95% confidence interval) associated with the *ADIPOR1 *genotypes were 0.53 (95% CI, 0.35-0.81) for rs12733285C/T, 0.59 (95% CI, 0.45-0.78) for rs1342387A/G, and 0.59 (95% CI, 0.39-0.89) for rs1342387A/A, respectively. Furthermore, the risks were more significant in carriers of the allele A of rs1342387A/G (adjusted OR, 0.59; 95% CI, 0.46-0.77) than noncarriers (G/G). In a further subgroup analysis, we observed that rs266729G/C was associated with an increased risk for colon cancer (adjusted OR, 1.50; 95% CI, 1.05-2.14) but not for rectal cancer (adjusted OR, 0.88; 95% CI, 0.63-1.22), and that carriers of the G allele had an increased risk for developing colon cancer (adjusted OR, 1.45; 95% CI, 1.03-2.05).

**Conclusions:**

We conclude that the rs12733285C/T genotype and the carriage of the A allele of rs1342387 (A/G or A/A) in *ADIPOR1 *are the protective factors for CRC, while that rs266729G/C and G allele of *ADIPOQ *are the risk factors for colon cancer after excluding rectal cancer cases.

## Background

Colorectal cancer (CRC) is one of the most common gastrointestinal tumors worldwide[1]. Epidemiological studies have demonstrated that some risk factors and interactions between genetic and environmental factors may play important roles in the pathogenesis of that cancer [2,3]. For example, individual genetic susceptibility is likely to play an important role in the development of some 35% of the CRC cases [4], suggesting that genetic background is one of the critical CRC risk factors [5,6].

Many studies have demonstrated that obesity could increase the risk of cancer, including CRC [7-9]. In obese patients, adiponectin, a protein secreted by adipose tissue, has lower expression than that in non-obese subjects [10,11], implying that decreased expression of adiponectin (*ADIPOQ*) may be associated with an increased risk for developing colorectal cancer (CRC) in humans [12-15]. Adiponectin and its receptors are expressed in colonic tissue [16]. For example, the expression of *ADIPOR1 *and *ADIPOR2 *are higher in colorectal carcinomas than in normal colonic epithelium [17]. Adiponectin seems to act in preneoplastic colonic lesions to regulate cell growth by activating, altering, or interacting with several pathways including leptin and NFκB pathway [18]. However, the opposite evidence has shown that adiponectin plasma levels are inversely associated with the risk of CRC [13], and that adiponectin can suppress the cell proliferation of colon cancer via AdipoR1- and -R2-mediated AMPK activation[19].

Several polymorphisms of adiponectin have been shown to affect adiponectin plasma levels[20,21]. Polymorphisms of the ligand (*ADIPOQ*,) and its type 1 receptor (*ADIPOR1*) are associated with the risk for insulin resistance, cardiovascular disease, and diabetes mellitus [20-26]. Polymorphisms of *ADIPOR1 *have been associated with the risk for cancers, probably by affecting adiponectin plasma levels [27-30]. Polymorphisms in the adiponectin gene have also been shown to correlate with adiponectin plasma levels, The genotypes rs266729 C/C, rs1501299 T/T, and rs2241766 G/G were respectively associated with decreased adiponectin plasma levels [20,21], suggesting that these polymorphisms may be associated with increased risk of developing CRC. In a population-based case-control study, we genotyped seven polymorphisms of *ADIPOQ *and *ADIPOR1 *genes to confirm such a hypothesis.

## Methods

Four hundred and twenty inpatients who had been diagnosed histologically as CRC (colon cancer, n = 191, and rectum cancer, n = 229) in Nanjing First hospital affiliated to Nanjing Medical University from January 2007 to January 2010 were recruited consecutively for this study. The case cohort ranged in age from 30 to 93 years (mean ± SD, 62.9 ± 12.3 years). Five hundred and fifty five age-and gender-matched healthy individuals who came to hospital for routine health check were employed as non-cancer controls whose ages ranged from 44 to 91 years (mean ± SD, 61.7 ± 10.7 years). Both case and control cohorts were from the same geographic region of Nanjing City, Jiangsu, China. For all CRC cases and control individuals, clinical characteristics of each subject, including smoking [Cumulative cigarette dose (pack-years) was calculated by the following formula: pack-years = (packs per day)×(years smoked), those who had smoked at least once a day for >1 year in their lifetime were considered smokers, and non-smokers were defined as ones who had stopped smoking at least 1 year before diagnosis in the patients and 1 year before the date signed on an informed consent for blood sample collection in the case of controls or who had never smoked in their lifetime.], drinking and related disease history (such as other cancer, diabetes and so on), was collected via a questionnaire designed in-house by the Department of Gastroenterology in the hospital. There was no significant difference in the demographic data between the two groups. This study protocol was approved by the Institutional Review Board of the hospital, and written informed consents were obtained from all participants.

Genomic DNA was extracted from peripheral blood mononuclear cells of CRC patients and controls using a E.Z.N.A.^® ^SE Blood DNA Kit (Omega Bio-Tek, Inc, Norcross, GA, UAS) according to the manufacturer's instructions. Briefly, cells were lysed by a cell lysis solution and contaminated RNA in the samples was then removed by RNase A treatment and the protein was precipitated by the protein precipitation solution. The genomic DNA was finally precipitated by isopropanol, followed by being washed with 70% ethanol.

Polymerase chain reaction-restriction fragment length polymorphism (PCR-RFLP) was applied to detect the polymorphic sites of *ADIPOQ *and *ADIPOR1 *as shown in Table 1. PCR amplification was performed in a 25 μL reaction system consisting of 0.5 mmol/L of each primer, 4 mmol/L of MgCl_2_, 1.25 U of Ampli Taq Polymerase, and PCR buffer (10 mmol/L of Tris-HCl, pH 8.3; 50 mmol/L of KCl). After the denaturation at 94°C 5 min, DNA amplification was achieved by 35 cycles of denaturing at 94°C for 30 sec, annealing at a certain temperature (as shown in Table 1) for 30 sec, extension at 72°C for 30 sec, and then followed by 72°C for 7 min. For restriction enzyme digestion, 10 μL PCR product was digested by 10 U of *Hin*6I (MBI Fermentas, Vilnius, Lithuania) with buffer Tango for rs266729, *Hinf *I (MBI) with buffer B for rs822395, *Ava*I (MBI) with buffer Tango for rs2241766, *Nsp*I(MBI) with buffer Tango for rs1501299, and *Nsi*I (MBI) with buffer R for rs12733285 in a final volume of 30 μL and incubated at 37°C(*Tru9 *I with buffer R for rs822396 at 65°C) overnight. Moreover, 10U *Bcc*I (New England Biolabs, Beverly, MA, USA) with NEBuffer 1 was utilized for digesting the 10 μL PCR product of rs1342387 in a final volume of 30 μL and incubated at 37°C overnight. Deoxyribonucleic acid fragments of rs266729, rs1342387 and rs822396 were separated by 3% agarose gel electrophoresis, and 4% agarose gel was used for the fragment of rs822395, rs2241766, rs2232853 and rs12733285. To validate the genotyping results, the genotyping experiments were repeated, a random selection of 10% of all samples was genotyped twice by direct sequence for quality control.

**Table 1:** Primers and PCR conditions for genotype detection of *ADIPOQ *and *ADIPOR1*

SNP	Gene	Position	Annual temperature	Primer sequence	Restriction enzyme	PCR-RFLP products(bp)	Genotype
				(5'-3')			
rs266729	*ADIPOQ*	5′ flanking -11365 C→G	63°C	GATGTCTTGTTGAAGTTGGTGCTG TAGAAAGTTTAGGCTTGAAGTGGC	*Hin6 *I	176 176, 100, 76 100, 76	CC GC GG
rs822395	*ADIPOQ*	Intron 1 -4034 A→C	50°C	TGATCGCACCTATTAGTGGAGgAAT* TCCAGAATATGTGAAAGCCCCAGAG	*Hinf *I	208 208, 187, 21 187, 21	AA AC CC
rs822396	*ADIPOQ*	Intron 1 -3964 A→G	58°C	ATCAGAGTCCGTTCTTGGT ACTGCTTGTCACCTCCACC	*Tru9 *I	458 458,259,199 259,199	GG GA AA
rs2241766	*ADIPOQ*	Exon 2 +45 T→G	51°C	GATCAAGGTGGGCTGCAATA AGTCGTGGTTTCCTGGTCAT	*Ava *I	246 246,220,26 220,26	TT TG GG
rs1501299	*ADIPOQ*	Intron 2 +276 G→T	50°C	TCTAGGCCTTAGTTAATAATGcA* CAGGAAACCACGACTCAAG	*Nsp *I	250 250,230,20 230,20	AA AC CC
rs12733285	*ADIPOR1*	Intron 1 -1742 C→T	66°C	GTCATGCTATGCTCAACCCACAtGC* CTTCCAGCTTCCTGTTCTCCTTAG	*Nsi *I	209 209,183,26 183,26	CC CT TT
rs1342387	*ADIPOR1*	Intron 4 +5843 G→A	60°C	CACCTGGTAGTGGGATTGG GAGAAGCTGAGGCAGAGCA	*Bcc *I	474 474,294,180 294,180	GG AG AA

* Primers in lower case letters denote the mismatched position that is required for subsequent restriction digestion for the differentiation of the polymorphisms.

Statistical analysis of genotype distribution and allele frequencies was performed by using the χ2 test with SPSS 11.0 for Windows (SPSS, Chicago, IL). Odds ratios (OR) and 95% confidence intervals (CIs) were calculated using a logistic regression model. Differences in mean values were evaluated using t-test. The *P *value < 0.05 was considered statistically significant.

## Results and Discussion

Four hundred twenty patients with colorectal cancer and five hundred fifty and five healthy controls were evaluated in this population-based case-control association study, and there were no statistically significant differences in their demographic data and clinical characteristics as summarized in Table 2.

**Table 2:** Demographic and clinical characteristics of CRC patients and the controls

Variables	Patients, n (%)	Controls, n (%)	P-value
Total cases	420	555	
Gender			
Male	280 (66.67)	339 (61.08)	0.084
Female	140(33.33)	216 (38.92)	
Age			
Mean ± SD	62.88 ± 12.32	61.71 ± 10.65	0.113
≤ 60	186(44.29)	271 (48.83)	0.082
>60	234(55.71)	284 (51.17)	
Smoking status (Pack-year)			
Non-smoker	316 (73.24)	406 (73.15)	0.508
Smoker	104(24.76)	149(26.85)	
Alcohol consumption (year)			
Never	274(65.24)	379 (68.29)	0.514
≤ 15	119 (28.33)	139(25.05)	
> 15	27 (6.43)	37 (6.67)	
Tumor site			
Colon	191(45.48)	--	--
Rectal	229(54.52)	--	

The genotype distribution of the polymorphisms of *ADIPOQ *and *ADIPOR1 *between the cases and controls are presented in Table 3. The observed frequencies of all tested genotypes and alleles in controls did not derivate from the Hardy-Weinberg equilibrium. Logistic regression analysis revealed a significantly decreased risk (adjusted OR, 0.53; 95% CI: 0.35-0.81) for the rs12733285 C/T heterozygote when compared with the rs12733285 C/C wild-type homozygote as shown in Table 3. Similarly, carriers of the A allele (rs1342387 A/G or A/A) had decreased risk (adjusted OR 0.59; 95% CI: 0.45-0.78, and adjusted OR 0.59; 95% CI: 0.39-0.89, respectively) for CRC compared with noncarriers (i.e., rs1342387 G/G) as shown in Table 3, suggesting that carriers of the rs1342387 A allele had a significantly decreased risk for colorectal cancer (adjusted OR 0.59; 95% CI: 0.46-0.77). In contrast, the distribution of the five polymorphisms (rs266729, rs822395, rs822396, rs1501766 and rs1501299) in *ADIPOQ *was not statistically significantly different between the case and control groups as shown in Table 3.

**Table 3:** The genotype distribution of *ADIPOQ *and *ADIPOR1 *in CRC patients and controls

Genotypes	Patients, n (%)	Controls, n (%)	OR (95% CI)^a^	OR (95% CI)^b^	*P *value^c^
rs266729					
CC	173(41.79)	243 (43.78)	Reference	Reference	0.705
GC	205 (48.81)	261(47.03)	1.10(0.84,1.44)	1.12(0.85,1.46)	
GG	42(10.00)	51(9.19)	1.16(0.74,1.82)	1.18(0.75,1.86)	
GC/GG	247(58.81)	312(56.22)	1.12(0.86,1.44)	1.13(0.87,1.46)	
rs822395					
AA	343(81.67)	440(79.28)	Reference	Reference	0.38
AC	70(16.67)	109(19.64)	0.82(0.59,1.15)	0.81(0.58,1.12)	
CC	7(1.67)	6(1.08)	1.50(0.50,4.49)	1.47(0.49,4.46)	
AC/CC	77(18.33)	115(20.72)	0.86(0.62,1.18)	0.84(0.61,1.16)	
rs822396					
AA	344(81.90)	450(81.08)	Reference	Reference	0.82
AG	73(17.38)	99(17.84)	0.97(0.69,1.35)	0.99(0.70,1.38)	
GG	3(0.71)	6(1.08)	0.65(0.16,2.63)	0.63(0.16,2.54)	
AG/GG	76(18.10)	105(18.92)	0.94(0.68,1.31)	0.96(0.69,1.34)	
rs2241766					
TT	190(45.24)	278(50.09)	Reference	Reference	0.265
TG	193(45.95)	238(42.88)	1.19(0.91,1.55)	1.18(0.91,1.54)	
GG	37(8.81)	39(7.03)	1.39(0.85,2.26)	1.42(0.86,2.32)	
TG/GG	230(54.76)	277(49.91)	1.22(0.94,1.57)	1.21(0.94,1.57)	
rs1501299					
CC	220(52.38)	265(47.75)	Reference	Reference	0.276
AC	160(38.10)	224(40.36)	0.86(0.66,1.13)	0.87(0.66,1.15)	
AA	40(9.52)	66(11.89)	0.73(0.47,1.12)	0.95(0.67,1.36)	
AC/AA	200(47.62)	290(52.25)	0.83(0.65,1.07)	0.84(0.65,1.09)	
rs12733285					
CC	386(91.90)	477(85.95)	Reference	Reference	0.004
CT	34(8.10)	78(14.05)	0.54(0.35,0.82)	0.53(0.35,0.81)	
TT	0(0.00)	0(0.00)	--	--	
CT/TT	34(8.10)	78(14.05)	0.54(0.35,0.82)	0.53(0.35,0.81)	
rs1342387					
GG	213(50.71)	210(37.84)	Reference	Reference	0
AG	157(37.38)	263(47.39)	0.59(0.44,0.78)	0.59(0.45,0.78)	
AA	50(11.90)	82(14.77)	0.60(0.40,0.90)	0.59(0.39,0.89)	
AG/AA	207(49.29)	345(62.16)	0.59(0.46,0.77)	0.59(0.46,0.77)	

^a^Crude odds ratio

^b^Age and gender adjusted odds ratio

^c^χ^2 ^test for all genotypes between the two groups

Subgroup analysis was performed based on the tumor site in this study, and logistic regression analysis revealed a significantly increased risk for the rs266729G/C heterozygote (adjusted OR, 1.50; 95% CI: 1.05-2.14) as compared with the rs266729 C/C wild-type homozygote as shown in Table 4, suggesting that carriers of the 266729 G allele had a significantly increased risk for colorectal cancer (adjusted OR 1.45; 95% CI: 1.03-2.05). However, there was no difference in the genotype distribution between the sub-cohort of colon cancer and rectal cancer.

**Table 4:** Genotype distribution in relation to sub sites in colorectal cancer

Genotypes	controls	Colon cancer		Rectal cancer	
			
	n (%)	n (%)	OR(95% CI)^a^	n(%)	OR (95% CI)^a^
rs266729					
CC	243(43.78)	68(35.60)	Reference	105(45.85)	Reference
GC	261(47.03)	107(56.02)	1.50(1.05,2.14)	98(42.79)	0.88(0.63,1.22)
GG	51(9.19)	16(8.38)	1.19(0.64,2.25)	26(11.35)	1.20(0.71,2.04)
GC/GG	312(56.22)	123(64.40)	1.45(1.03, 2.05)	124(54.15)	0.94(0.69,1.28)
rs822395					
AA	440(79.28)	155(81.15)	Reference	188(82.10)	Reference
AC	109(19.64)	32(16.75)	0.82(0.53,1.27)	38(16.59)	0.80(0.53,1.20)
CC	6(1.08)	4(2.09)	1.63(0.45,5.97)	3(1.31)	1.25(0.31,5.06)
AC/CC	115(20.72)	36(18.85)	0.86(0.56,1.31)	41(17.90)	0.82(0.55,1.22)
rs822396					
AA	450(81.08)	161(84.29)	Reference	183(79.91)	Reference
AG	99(17.84)	28(14.66)	0.830(0.52,1.31)	45(19.65)	1.11(0.75,1.65)
GG	6(1.08)	2(1.05)	0.93(0.18,4.70)	1(0.44)	0.38(0.05,3.17)
AG/GG	105(18.92)	30(15.71)	0.83(0.53,1.30)	46(20.09)	1.07(0.72,1.58)
rs2241766					
TT	278(50.09)	91(47.64)	Reference	99(42.23)	Reference
TG	238(42.88)	87(45.55)	1.10(0.78,1.56)	106(46.29)	1.25(0.90,1.72)
GG	39(7.03)	13(6.81)	1.07(0.54,2.11)	24(10.48)	1.71(0.97,3.01)
TG/GG	277(49.91)	100(52.36)	1.10(0.79,1.53)	130(56.77)	1.31(0.96.1.79)
rs1501299					
CC	265(47.75)	97(50.79)	Reference	123(53.71)	Reference
AC	224(40.36)	72(37.70)	0.86(0.60,1.24)	88(38.43)	0.88(0.63,1.22)
AA	66(11.89)	22(11.52)	0.93(0.54,1.60)	18(7.86)	0.59(0.34,1.04)
AC/AA	290(52.25)	94(49.21)	0.89(0.64,1.24)	106(46.29)	0.81(0.59,1.11)
rs12733285					
CC	477(85.95)	175(91.62)	Reference	211(92.14)	Reference
CT	78(14.05)	16(8.38)	0.51(0.29,0.91)	18(7.86)	0.51(0.30,0.88)
TT	0(0.00)	0(0.00)	--	0(0.00)	--
CT/TT	78(14.05)	16(8.38)	0.51(0.29,0.91)	18(7.86)	0.51(0.30,0.88)
rs1342387					
GG	210(37.84)	99(51.83)	Reference	114(49.78)	Reference
AG	263(47.39)	69(36.13)	0.57(0.40,0.81)	88(38.43)	0.62(0.45,0.87)
AA	82(14.77)	23(12.04)	0.54(0.32,0.93)	27(11.79)	0.66(0.40,1.09)
AG/AA	345(62.16)	92(48.17)	0.57(0.41,0.79)	115(50.22)	0.62(0.46,0.85)

^a^Age and gender adjusted odds ratio

Through the population-based case-control study of 420 CRC patients and 555 age-and gender- matched healthy controls in the Chinese population, we observed that CRC patients had lower frequency of rs2733285C/T and rs1342387A/G or A/A than healthy controls, and that carriers of the rs2733285T and rs1342387A allele had a significantly decreased risk for developing CRC. Moreover, we also revealed that rs266729G/C genotype and rs266729G allele was a risk factor for colon cancer.

It has been well known that obesity is one of the major risks for CRC [31,32] and that lack of physical exercise, diets with high sugar, refined grains, and low fiber are all believed as the risks of CRC [33-36]. Adiponectin is secreted by adipose tissue, and epidemic studies have demonstrated that its plasma levels were inversely correlated to BMI [37,38]. Recent studies have shown that polymorphism in *ADIPOQ *(rs2241766) is associated with the risk of breast cancer [27], and that a polymorphism rs266729 is associated with the risk of colorectal cancer [28]. However, in this case-control study, we failed to replicate the association between the two positive variants at *ADIPOQ *(rs2241766, and rs266729) and CRC risk in Chinese population. Our findings are consistent with the studies reported in Czech [30] and British populations[39]. However, our subgroup analysis still revealed that rs266729 G/C and 266729 G allele were risk factors for colon cancer but not for rectal cancer. These findings may partly explain the previous contradictive studies on the association between the polymorphism of rs2241766 and CRC risk [28]. Moreover, basic research had also predicted that *ADIPOQ *and *ADIPOR *have more potential effects on colon cancer than on rectal cancer [40], indicating that rs266729 G/C and 266729 G allele were risk factors for colon cancer but not for rectal cancer, several studies have confirmed that polymorphisms rs266729 C/C, rs1501299 T/T and rs2241766 G/G in the *ADIPOQ *were associated with adiponectin plasma levels [20,21,24]. In particularly, such association were also deduced in Chinese population that G allele of rs266729 was significantly associated with lower adiponectin plasma levels[41], and that low levels of adiponectin was associated with the increased risk of CRC [13,42].

The association between adiponectin type 1 receptor level and the two polymorphisms (rs12733285, rs1342387) in *ADIPOR *was still unclear. In this case-control study, the association between polymorphisms in *ADIPOR1 *(rs12733285, rs1342387) and CRC risk has also been investigated, and the results indicate that individuals with rs12733285C/T or rs1342387A/G or A/A had a decreased risk of CRC, which was similar with the finding in North American populations [28]. However, we have not observed the rs12733285T/T genotype both in case and control groups, in a sharp contrast with 15% of the rs12733285T/T genotype in North American population [27,28]. Nevertheless, our results are consistent with Hap-Map data among western Asian population http://www.hapmap.org/index.html.en. For polymorphism (rs1342387) of *ADIPOR1*, the distribution of genotypes in our studies was similar to the results by Wang et al [43]. Previous clinical studies have shown that the genotypes rs1342387A/G or A/A were associated with higher adiponectin plasma levels [21], and G/G genotype of rs1342387 of the *ADIPOR1 *gene was also associated with the indicators of obesity [44,45]. Therefore, the reason of the G/G genotype of rs1342387 as a risk factor for CRC may be correlated to obesity and insulin resistance. In addition, clinical studies have also revealed that adiponectin type 1 receptor is related to colorectal cancer progression[46]. However, the function of rs1342387 in *ADIPOR1 *remains unclear, and the direct relationship between rs1342387 and the risk of CRC needs to be further evaluated.

## Conclusions

This case-control study demonstrates that polymorphisms in *ADIPOR1 *(rs12733285, rs1342387) are associated with the decreased risk of CRC, and polymorphism in *ADIPOQ *(rs266729) is a risk for colon cancer but not for rectal cancer.

## List of abbreviations

ADIPOQ: adiponectin; CRC: colorectal cancer; ADIPOR1: adiponectin type 1 receptor (); OR: odds ratio; CI: confidence interval

## Competing interests

The authors declare that they have no competing interests.

## Authors' contributions

BH, YZ, QB participated in the genotype detection of samples. LC, ZN, LG and YX collected the samples and clinical characteristics of each subject. BH and YP designed the study and performed the statistical analysis and involved in drafting the manuscript or revising it critically for important intellectual content, SW conceived of the study, and participated in its design and coordination. All authors read and approved the final manuscript.

## Pre-publication history

The pre-publication history for this paper can be accessed here:

http://www.biomedcentral.com/1471-2350/12/94/prepub
